# Outlines of Military Surgery

**Published:** 1845-07

**Authors:** 


ART. II.—*Outlines of Military Surgery*. By SIR GEORGE BALLINGALL, M.D. F.R.S.E. &c. Third Edition.—*Edinburgh*, 1844. 8vo, pp. 568.

IT may be considered a sufficient testimony to the utility of these Outlines, and to the able manner in which they have been executed, that a third edition has been called for. Sir George Ballingall has taken the opportunity to add to the work and, as we think, materially to improve it. This is most evident in that part which treats of the diseases of troops on foreign stations, which he has illustrated by reference to the 'Statistical Reports on the Health of the Navy and Army,' although he has perhaps not drawn upon these so liberally as their importance and indisputable authenticity would have warranted. We are surprised to find that he has not in any instance referred to the Statistics of the United States Army.

The principal part of the work is devoted to the consideration of "those surgical accidents and diseases peculiarly incident to military and naval men; and to which the nature of their profession exposes them in all quarters of the world." In the execution of this part our author has evidently taken great pains to obtain the most recent information on the subjects he treats of, and has discussed in a very temperate manner the various *questiones vexatæ*, giving due weight to the opinions of those with whom he differs.

We could have wished, however, that he had curtailed the portion of his volume which treats of surgery, and extended that embracing what John Bell termed military economics. On the former subject we have numerous works, as those of Liston, Fergusson, Syme, Druitt, &c., while on the latter a good one in the English language is still a desideratum. As a reason for the plan he has adopted, our author expresses a fear "that in a period of long protracted peace there is some risk of the medical officers of the army overlooking the importance of the surgical department of their profession." In this fear we do not participate; the éclat attending successful operations has ever led a large proportion of students to devote themselves to surgery, while the less imposing but no less arduous duties of the physician, have been looked on as very subordinate matters. We venture to assert that many more will at all times be found to devote themselves to surgical practice than to that untiring watchfulness and patient superintendence, which is of so much importance in those to whom the health of a body of troops is confided.

Of the great value of this part of a medical officer's duty on service, Mr. Guthrie gives the following most striking example:

"I remember a village on the great plain of the Guadiana, near Merida, in which there regiments were quartered in the sickly season in the autumn, when fever prevails. Three rows of hillocks marked the last resting place of the dead on earth, and my attention was attracted by one row being much shorter than the other two. I found on inquiry that the regiments were very much of the same strength, and quite under the same circumstances. The doctors were equally able: two were men entering on rather the middle period of life, the third was a very young man and perhaps the worst doctor of the three; but the short row of tumuli belonged to him. I was very desirous of making this out, and after carefully visiting all the hospitals and quarters ascertained the reason. He was the better soldier, if not the best doctor. His hospitals were in better order, the material was more perfect, the labour bestowed on every part, except in physic, was greater, and five per cent. at least of human life was the saving and the result. I never saw it otherwise." (Clinical Lectures, p. 20.)

We have made these remarks in the hope that when another edition is required, our author will devote a greater space to the consideration of the various means necessary "for the prevention as well as the cure of disease; for it is by prevention rather than by cure that the efficiency of our fleets and armies is to be maintained." The present volume as a work on Military Surgery will add to the already high reputation of the author, and should form part of every military and naval medical officer's library. It will, however, we think, be more appreciated by gentlemen on, or returning from, foreign service, who wish "to refresh their memories or renovate their surgical knowledge," than by young men commencing their career in the public service; to the latter, the addition of a practical treatise on military economics would be a valuable acquisition.

